# Machine learning-based risk prediction of overt hepatic encephalopathy after transjugular intrahepatic portosystemic shunt in patients with cirrhosis: a cohort study

**DOI:** 10.3389/fmed.2026.1809634

**Published:** 2026-05-29

**Authors:** Lixin Song, Xinyi Qiao, Long Gao, Yu Sun, Duiping Feng, Hui Yang

**Affiliations:** 1 Academy of Medical Sciences, Shanxi Medical University, Taiyuan, Shanxi, China; 2 The First Clinical Medical College, Shanxi Medical University, Taiyuan, Shanxi, China; 3 Department of Oncological and Vascular Intervention, The First Hospital of Shanxi Medical University, Taiyuan, Shanxi, China; 4 Department of Infectious Diseases, The First Hospital of Shanxi Medical University, Taiyuan, Shanxi, China

**Keywords:** hepatic encephalopathy, machine learning, portal hypertension, risk factors, transjugular intrahepatic portosystemic shunt

## Abstract

**Background:**

Overt hepatic encephalopathy (OHE) is a frequent complication after transjugular intrahepatic portosystemic shunt (TIPS) in patients with cirrhosis and can markedly impair quality of life and prognosis. This study aimed to develop and validate a machine learning-based risk prediction model to determine the most effective model and key predictive factors.

**Methods:**

This retrospective study included 297 patients with cirrhosis who underwent TIPS at the First Hospital of Shanxi Medical University from 2019 to 2024, among whom 89 developed postoperative OHE. Preoperative clinical characteristics and procedure-related variables were compared between the OHE and non-OHE groups using univariate analyses. Feature selection was conducted using least absolute shrinkage and selection operator regression and random forest. The selected variables were then used to develop five machine learning models: logistic regression, support vector machine, random forest, extreme gradient boosting (XGBoost), and artificial neural network. Model performance was evaluated in terms of discrimination, calibration, and clinical utility. The optimal model was further interpreted using Shapley additive explanations to identify key predictors.

**Results:**

The final predictors incorporated into the model were BUN, GGT, Age, FIB, and the portal vein puncture site. Model comparisons indicated some variation in predictive performance across models in the test cohort. XGBoost achieved an AUC of 0.792 (95% CI: 0.671–0.914) and showed relatively stable performance in calibration and clinical net benefit.

**Conclusion:**

The XGBoost model was developed using routine clinical indicators and procedure-related factors. It demonstrates potential utility in estimating the risk of post-TIPS OHE and may serve as an adjunct in preoperative risk assessment.

## 1 Introduction

Transjugular intrahepatic portosystemic shunt (TIPS) is a well-established intervention for the management of esophageal variceal bleeding and refractory ascites. By establishing a stent-supported intrahepatic channel between the hepatic and portal veins, TIPS effectively reduces portal venous pressure (1, 2). Nevertheless, postoperative complications still limit its broader utilization, with hepatic encephalopathy (HE) being among the most common. HE typically occurs within 1 month to 1 year after TIPS, with an incidence ranging from 25 to 50% (3, 4). It significantly compromises patients’ quality of life and survival outcomes. HE is classified as covert (CHE) or overt (OHE) according to the West Haven criteria. OHE corresponds to grades 2–4 and is characterized by impaired consciousness and neuropsychiatric abnormalities. It is a major complication after TIPS. Therefore, identifying patients at high risk of OHE before the procedure and determining the associated risk factors is important for optimizing perioperative management and guiding individualized preventive strategies.

Current research on predicting OHE after TIPS has largely relied on traditional statistical approaches, such as multivariable logistic regression and Cox regression, with individualized risk assessment typically presented through nomograms (5, 6). However, these models are constrained by linear assumptions and have limited ability to capture potential nonlinear relationships among variables, which may affect predictive performance and generalization. In recent years, machine learning (ML) has advanced rapidly in the field of medical data analysis. It offers the ability to identify nonlinear relationships, handle high-dimensional features, and improve predictive accuracy. Accordingly, it has been widely applied in disease prediction and prognostic assessment (7). Therefore, the application of machine learning to predicting OHE after TIPS has meaningful research and clinical value.

In this study, five ML models were constructed, comprising logistic regression (LR), support vector machine (SVM), random forest (RF), extreme gradient boosting (XGBoost) and artificial neural network (ANN), to predict the risk of OHE after TIPS in patients with cirrhosis. The optimal model was identified through a systematic comparison of discrimination, calibration, and clinical net benefit. Furthermore, key predictive factors were determined based on feature importance analyses, providing a basis for preoperative risk assessment and individualized decision-making in TIPS candidates.

## 2 Materials and methods

### 2.1 Research design and ethics

This retrospective study collected clinical data from patients with liver cirrhosis who underwent TIPS procedures at the First Hospital of Shanxi Medical University between 2019 and 2024. This study was approved by the Ethics Committee of the First Hospital of Shanxi Medical University (approval number: NO. KYLL-2026-108), and the requirement for informed consent was waived due to its retrospective design. Because this study included all eligible cases during the study period, no *a priori* sample size calculation was performed. The number of included outcome events provided a reasonable basis for model development.

### 2.2 Data collection and inclusion/exclusion criteria

#### 2.2.1 Data collection

Collected variables encompassed general clinical characteristics, including sex, age, etiology of cirrhosis, diabetes, and prior history of HE, as well as TIPS indications, namely gastrointestinal bleeding and refractory ascites. Preoperative serological parameters included blood ammonia (NH₃), creatinine (Cr), serum sodium (Na), international normalized ratio (INR), total bilirubin (TBiL), blood urea nitrogen (BUN), alkaline phosphatase (ALP), gamma–glutamyl transferase (GGT), prealbumin (PA), albumin (ALB), prothrombin time (PT), and fibrinogen (FIB). Additional clinical assessments comprised ascites, albumin–bilirubin (ALBI) score, Child-Pugh score, and MELD-Na score. Procedure-related variables included the portal vein puncture site (left or right branch), and pre- and postoperative portal venous pressures. All the relevant clinical information about the study population was extracted from the hospital’s electronic medical records system.

#### 2.2.2 Inclusion and exclusion criteria

Inclusion criteria: (1) A diagnosis of liver cirrhosis, as defined in the Chinese consensus on clinical diagnosis and therapy of liver cirrhosis; (2) Hospitalized patients with liver cirrhosis who fulfilled the TIPS indications outlined in the CCI clinical practice guidelines: Management of TIPS for Portal Hypertension (2019 edition) and subsequently underwent the procedure were included in the study; and (3) A diagnosis of HE consistent with Chinese guidelines on the management of hepatic encephalopathy in cirrhosis (2024). Exclusion criteria: (1) Presence of primary or secondary hepatocellular carcinoma or any other malignant neoplasm; (2) Severe dysfunction of the cardiac, pulmonary, renal, or other major organ systems; (3) Loss to follow-up within 3 months after undergoing TIPS; and (4) Patients with more than 20% missing data.

#### 2.2.3 Outcome definition

In this study, follow-up was performed through telephone contact or routine outpatient visits to ascertain whether OHE occurred within 1 year after TIPS. The first occurrence of OHE during the follow-up period was defined as the outcome. All episodes of OHE were assessed by the relevant clinical physicians according to the West Haven criteria.

#### 2.2.4 Post-TIPS management

Post-TIPS management at our center included routine prophylaxis for hepatic encephalopathy. Patients received oral L-ornithine–L-aspartate granules, 3 g three times daily, and lactulose 10–20 mL twice daily (with the dose adjusted according to bowel movements). These therapies were part of a standardized postoperative protocol and were applied consistently across all patients in the cohort.

### 2.3 Study procedures

All data processing and analyses were conducted using R software (version 4.3.3).

#### 2.3.1 Data preprocessing

A missing-value analysis was performed on the original dataset. Variables with more than 20% missingness were excluded. For variables with less than 20% missingness, the missingness mechanism was assessed and, assuming it satisfied the Missing at Random condition, missing values were imputed using the random forest–based missForest algorithm. This approach was employed to reduce bias associated with complete-case deletion and to preserve the underlying relationships among variables. The dataset was then divided into training and test cohorts at an 8:2 ratio. Given the differing dependence of the models on feature scaling, a differentiated standardization strategy was applied. For SVM and ANN, continuous variables in the training cohort were standardized using Z-score transformation, and the same parameters were applied to the test cohort to avoid information leakage. LR, RF and XGBoost were trained using the original, non-standardized features.

#### 2.3.2 Statistical analysis

Categorical variables were summarized as counts and percentages, and group comparisons were conducted using the chi-squared test or Fisher’s exact test. Continuous variables that followed a normal distribution were reported as the mean ± standard deviation (SD) and compared using an independent samples *t*-test. Non-normally distributed continuous variables were presented as the median and interquartile range (IQR) and compared using the Mann–Whitney U test. All analyses were two-sided, and *p* < 0.05 was considered statistically significant.

#### 2.3.3 Feature selection

To ensure stable and consistent feature selection, Least Absolute Shrinkage and Selection Operator (LASSO) regression and random forest methods were applied to the training cohort using the glmnet and randomForest packages in R. The optimal penalty parameter (*λ*) in the LASSO regression model was determined using 10-fold cross-validation, and variables with non-zero coefficients were selected. In the random forest model, variable importance was ranked by the Mean Decrease Gini, and predictors with higher contributions were retained. The final predictors included in the model were established by integrating the results of both methods and incorporating clinical relevance.

#### 2.3.4 Model development and evaluation

The selected variables were incorporated into LR, SVM, RF, XGBoost, and ANN models for training. All models were developed using the training cohort and hyperparameter tuning was performed through five-fold cross-validation. After model training, Platt scaling was fitted to the raw predicted probabilities from the training cohort and subsequently applied to both the training and test cohorts to obtain calibrated probabilities. Platt scaling is a post-hoc calibration method based on LR that improves the agreement between predicted probabilities and the observed event risk (8).

We evaluated the discriminatory performance of each model using the area under the receiver operating characteristic (ROC) curve, as well as sensitivity, specificity, the F1 score, and balanced accuracy. Because the dataset was imbalanced, overall accuracy does not adequately reflect performance in the minority class. Balanced accuracy was therefore used as a more appropriate summary measure. A fixed probability threshold of 0.40 was applied for binary classification to provide a more balanced compromise between sensitivity and specificity in the training cohort. Pairwise comparisons of the area under the curve (AUC) were performed using DeLong’s test to evaluate differences in model discrimination within the test cohort. Calibration performance was assessed using calibration curves together with the Hosmer–Lemeshow (H–L) goodness of fit test. Clinical utility was evaluated through decision curve analysis (DCA). Additionally, shapley additive explanations (SHAP) was used to evaluate the contribution and direction of effect of individual features in the model with the highest overall performance.

## 3 Results

**Figure 1:**
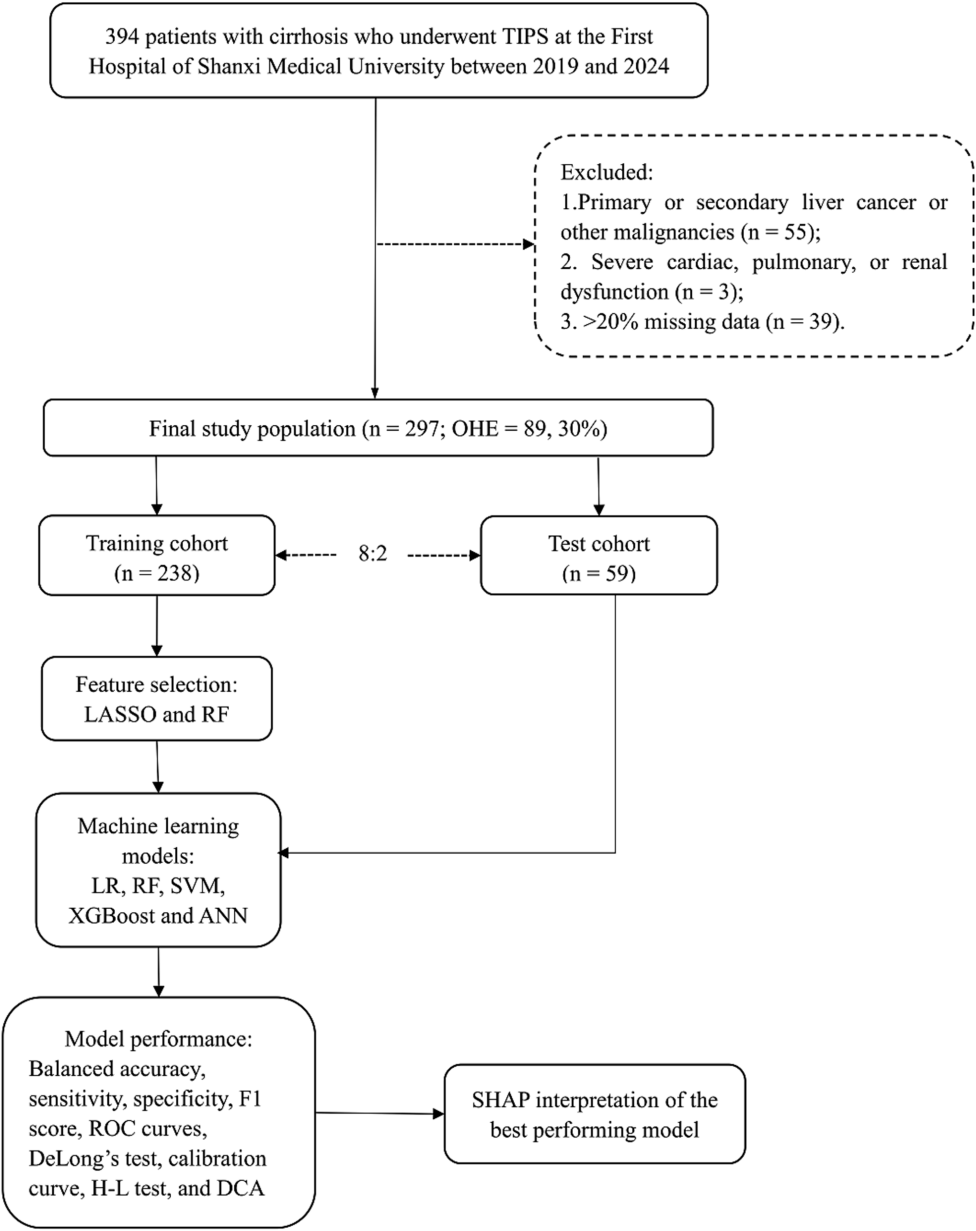
The flowchart of complete study.

A total of 394 patients who underwent TIPS at the First Hospital of Shanxi Medical University between 2019 and 2024 were initially screened. Of these, 297 patients were ultimately included in the analysis according to the inclusion and exclusion criteria (Figure 1).

### 3.1 Baseline characteristics

The present study comprised 297 patients with cirrhosis who underwent TIPS, of whom 164 were male and 133 female. OHE occurred in 89 patients within 1 year after the procedure, corresponding to an incidence of 30% (Figure 1). 96 patients (32.3%) had at least one missing baseline value. Missingness involved the following variables: PA (17.5%), NH_3_ (13.8%), GGT (9.4%), ALP (5.4%), BUN (2.0%), FIB (1.7%), Na (1.0%), Cr (0.3%), INR (0.3%), TBiL (0.3%), ALB (0.3%), and PT (0.3%). All missing data were imputed.

Univariate analyses were performed to compare variables between the OHE and non-OHE groups, with *p* ≤ 0.05 considered statistically significant. The results indicated that both Age and the portal vein puncture site were associated with the development of post-TIPS OHE (Table 1).

**Table 1:** Comparison of patient characteristics between the OHE and non-OHE groups.

Characteristics	Overall (*n* = 297)	OHE (*n* = 89)	Non-OHE (*n* = 208)	Test statistic (χ^2^/t/z)	*P* value
Sex (*n* %)				0.008	0.928
Male	164 (55.2)	50 (56.2)	114 (54.8)		
Female	133 (44.8)	39 (43.8)	94 (45.2)		
Age (years)	56.46 ± 10.93	59.16 ± 10.44	55.31 ± 10.95	−2.864	0.005
Etiology of cirrhosis (*n* %)				3.704	0.593
Hepatitis B	109 (36.7)	29 (32.6)	80 (38.5)		
Hepatitis C	18 (6.1)	4 (4.5)	14 (6.7)		
Alcoholic cirrhosis	53 (17.8)	18 (20.2)	35 (16.8)		
Autoimmune cirrhosis	39 (13.1)	12 (13.5)	27 (13.0)		
Cholestatic cirrhosis	34 (11.4)	14 (15.7)	20 (9.6)		
Others	44 (14.8)	12 (13.5)	32 (15.4)		
Prior history of HE (*n* %)				0.140	0.708
Yes	13 (4.4)	5 (5.6)	8 (3.8)		
No	284 (95.6)	84 (94.4)	200 (96.2)		
Diabetes (*n* %)				3.405	0.065
Yes	62 (20.9)	25 (28.1)	37 (17.8)		
No	235 (79.1)	64 (79.1)	171 (82.2)		
Ascites (*n* %)				0.986	0.611
None	91 (30.6)	26 (29.2)	65 (31.2)		
Mild to moderate	171 (57.6)	50 (56.2)	121 (58.2)		
Large volume	35 (11.8)	13 (14.6)	22 (10.6)		
Indication for TIPS (*n* %)				0.017	0.896
Gastrointestinal bleeding	271 (91.2)	82 (92.1)	189 (90.9)		
Refractory ascites	26 (8.8)	7 (7.9)	19 (9.1)		
Laboratory parameters					
NH_3_ (umol/L)	27.90 (16.90–39.10)	31.00 (16.60–43.90)	26.00 (16.90–37.38)	−1.705	0.088
Cr (umol/L)	61.50 (53.20–74.00)	61.00 (50.30–72.10)	62.00 (54.20–74.45)	0.698	0.486
Na (mmol/L)	140.00 (137.00–142.00)	139.00 (137.00–142.00)	140.00 (137.00–142.00)	0.197	0.844
TBiL (umol/L)	23.10 (15.70–34.90)	23.10 (15.30–34.00)	23.55 (16.10–35.42)	0.256	0.799
ALB (g/L)	32.35 ± 4.74	32.12 ± 4.08	32.45 ± 5.00	0.594	0.554
BUN (mmol/L)	5.96 (4.33–8.63)	6.03 (4.44–8.80)	5.92 (4.26–8.49)	−0.795	0.427
PA (mg/L)	115.00 (93.00–141.00)	115.00 (96.00–136.00)	115.00 (93.00–142.00)	0.492	0.623
ALP (U/L)	80.00 (62.00–112.00)	84.00 (64.00–133.00)	78.00 (61.75–109.00)	−1.161	0.246
GGT (U/L)	34.00 (20.00–60.00)	40.00 (26.00–67.00)	33.00 (19.00–56.25)	−1.858	0.063
INR	1.32 (1.21–1.51)	1.31 (1.18–1.48)	1.33 (1.22–1.51)	1.045	0.296
FIB (g/L)	1.70 (1.34–2.11)	1.75 (1.35–2.27)	1.69 (1.34–2.07)	−1.055	0.292
PT (s)	16.30 (14.40–18.80)	16.20 (14.10–18.10)	16.30 (14.40–19.20)	1.126	0.260
Procedural factors					
Pre-TIPS portal pressure (mmHg)	32.00 (28.00–35.00)	33.00 (29.00–34.00)	32.00 (28.00–35.00)	−0.554	0.579
Post-TIPS portal pressure (mmHg)	22.00 (18.00–25.00)	23.00 (19.00–25.00)	21.00 (18.00–25.00)	−1.245	0.213
Portal vein puncture site (*n* %)				9.800	0.002
Left branch	182 (61.3)	42 (47.2)	140 (67.3)		
Right branch	115 (38.7)	47 (52.8)	68 (32.7)		
Risk scoring systems					
Child-Pugh	8.00 (6.00–9.00)	8.00 (7.00–9.00)	8.00 (6.00–9.00)	0.143	0.885
MELD-Na	12.00 (10.00–15.00)	12.00 (10.00–15.00)	12.00 (10.00–15.00)	0.470	0.638
ALBI	−1.83 ± 0.47	−1.82 ± 0.42	−1.84 ± 0.49	−0.322	0.748

### 3.2 Feature selection

**Figure 2:**
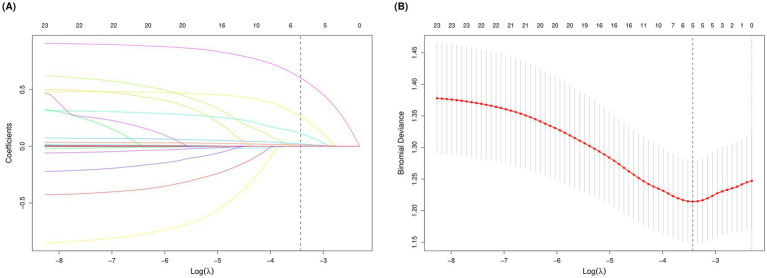
Feature selection. **(A)** The LASSO coefficient values profiles of variables. **(B)** The optimal lambda in the LASSO regression.

**Figure 3:**
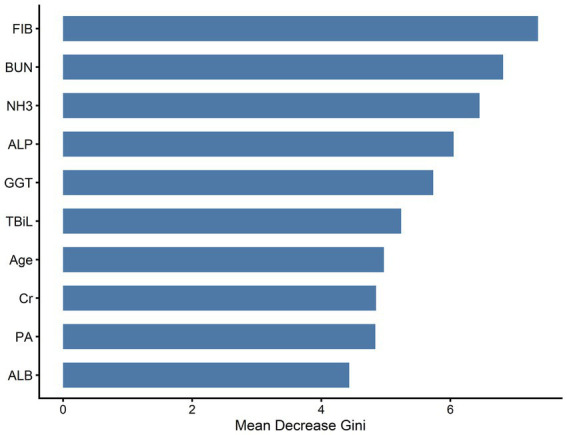
Importance ranking of predictors in the random forest model.

Under the optimal *λ*, the LASSO regression model retained five variables with non-zero coefficients: Age, diabetes, FIB, BUN, and the portal vein puncture site (Figure 2). In the random forest model, variable importance was assessed using the Mean Decrease Gini, which ranked FIB, BUN, NH₃, ALP, GGT, TBiL, Age, Cr, PA, and ALB as the top ten contributing features (Figure 3). After integrating the results from both LASSO regression and random forest analyses and considering clinical relevance, five predictors were selected for the final model: Age, BUN, portal vein puncture site, FIB and GGT.

### 3.3 Model performance

#### 3.3.1 Discriminative performance

**Figure 4:**
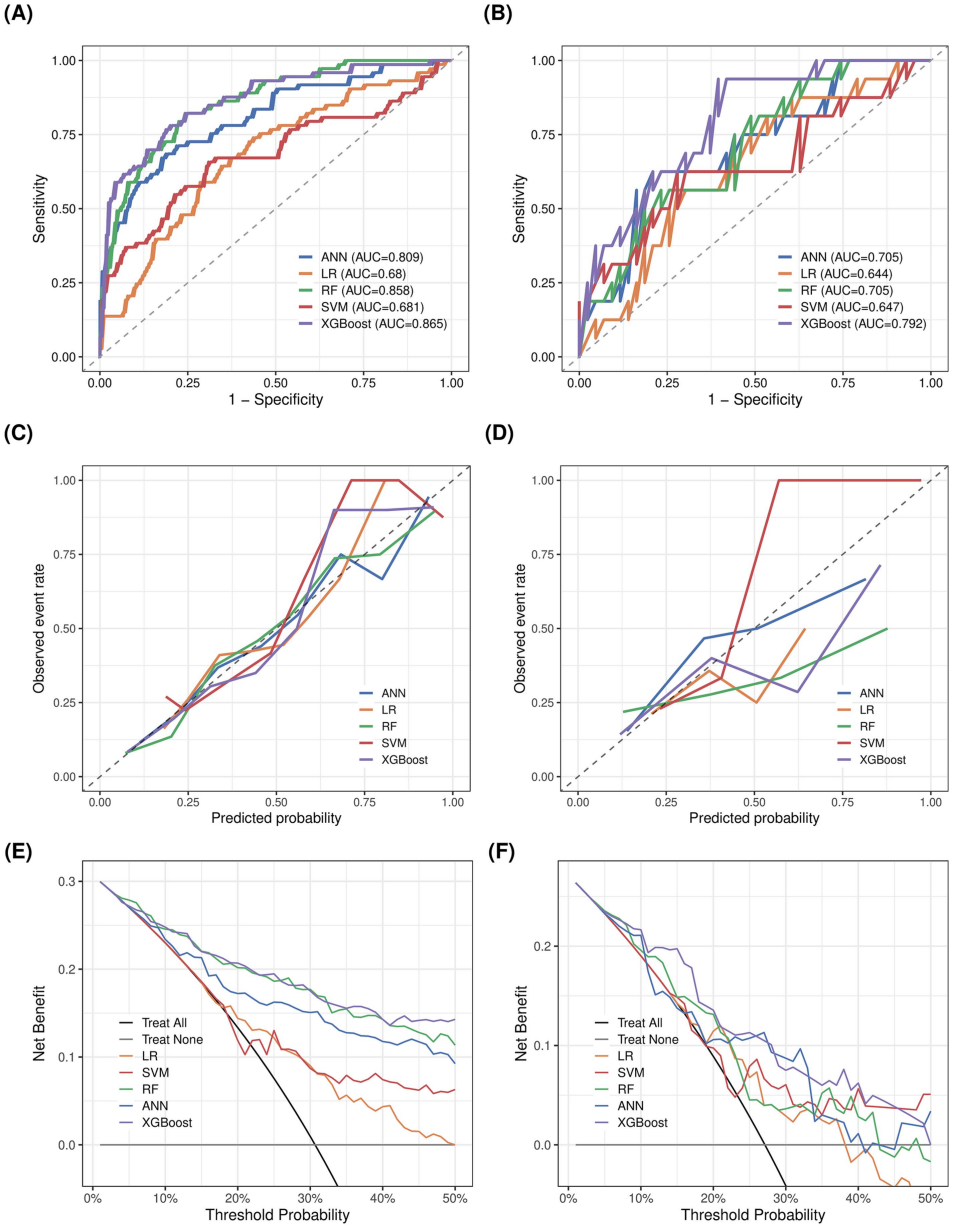
Performance comparison of different ML models in training cohort and test cohort. **(A)** ROC curves in the training cohort. **(B)** ROC curves in the test cohort. **(C)** Calibration curves in the training cohort. **(D)** Calibration curves in the test cohort. **(E)** DCA curves in the training cohort. **(F)** DCA curves in the test cohort.

In the training cohort, AUC values for the LR, SVM, RF, XGBoost and ANN models were 0.680 (95% CI: 0.605–0.754), 0.681 (95% CI: 0.600–0.763), 0.858 (95% CI: 0.808–0.908), 0.865 (95% CI: 0.814–0.917) and 0.809 (95% CI: 0.747–0.872), respectively. In the test cohort, the corresponding AUCs were 0.644 (95% CI: 0.488–0.800), 0.647 (95% CI: 0.471–0.823), 0.705 (95% CI: 0.560–0.850), 0.792 (95% CI: 0.671–0.914) and 0.705 (95% CI: 0.554–0.856). Notably, the XGBoost model achieved the highest AUC in both the training and test cohorts (Figures 4A,B). In terms of other performance metrics, XGBoost showed higher balanced accuracy (0.688) and sensitivity (0.563), whereas SVM showed the highest specificity (0.977) (Table 2).

**Table 2:** Performance of ML models in training cohort and test cohort.

Models	AUC (95% CI)	Balanced accuracy	Sensitivity	Specificity	F1 score
Training cohort					
LR	0.680 (0.605–0.754)	0.614	0.397	0.830	0.466
SVM	0.681 (0.600–0.763)	0.638	0.342	0.933	0.459
RF	0.858 (0.808–0.908)	0.769	0.685	0.855	0.680
XGBoost	0.865 (0.814–0.917)	0.765	0.658	0.873	0.676
ANN	0.809 (0.747–0.872)	0.729	0.603	0.855	0.624
Test cohort					
LR	0.644 (0.488–0.800)	0.571	0.375	0.767	0.375
SVM	0.647 (0.471–0.823)	0.613	0.250	0.977	0.381
RF	0.705 (0.560–0.850)	0.626	0.438	0.814	0.452
XGBoost	0.792 (0.671–0.914)	0.688	0.563	0.814	0.545
ANN	0.705 (0.554–0.856)	0.567	0.250	0.884	0.320

DeLong’s test for AUCs in the test cohort demonstrated a significant difference between XGBoost and LR (*p* = 0.038), whereas no significant differences were identified between XGBoost and the remaining models (Table 3). Sensitivity analyses based on two alternative feature sets (Supplementary Tables S1, S2) further indicated that the overall discriminatory performance and the relative ranking of the models remained consistent. Additionally, to further assess performance robustness, we conducted a 5-fold repeated cross-validation (10 repetitions) on the full dataset. The mean AUC ± SD for each model is presented in Supplementary Table S3, and the results were in line with the patterns observed in the primary analysis. Overall, XGBoost showed stable performance across metrics. However, the available evidence does not indicate a statistically significant advantage over the other models.

**Table 3:** Pairwise comparisons of AUCs for ML models in the test cohort using DeLong’s test.

Comparison	*P* value
LR vs. SVM	0.971
LR vs. RF	0.203
LR vs. XGBoost	0.038
LR vs. ANN	0.337
SVM vs. RF	0.452
SVM vs. XGBoost	0.077
SVM vs. ANN	0.486
RF vs. XGBoost	0.056
RF vs. ANN	1.000
XGBoost vs. ANN	0.216

#### 3.3.2 Calibration performance

The calibration curves for all models in both the training and test cohorts are shown in Figures 4C,D. In the training cohort, predicted and observed risks aligned reasonably well across models. In contrast, the test cohort demonstrated greater variability, with several models showing visible departures from the reference line. For the XGBoost model specifically, the calibration intercept was −0.289 and the slope was 0.707, indicating systematic underestimation and some degree of over-shrinkage in the predicted probabilities. Although the H–L test (χ^2^ = 7.879, df = 8, *p* = 0.445) did not indicate lack of fit, the calibration plot revealed noticeable deviations from the ideal line, most evidently at higher predicted risk levels.

#### 3.3.3 Clinical utility

The DCA showed that the XGBoost model achieved higher net benefit within the clinically relevant threshold probability range of 0.10–0.35. Although its net benefit declined at higher thresholds, the curve remained consistently above the treat-none line across all thresholds, indicating a potential advantage over the other models (Figures 4E,F).

#### 3.3.4 Model interpretability

**Figure 5:**
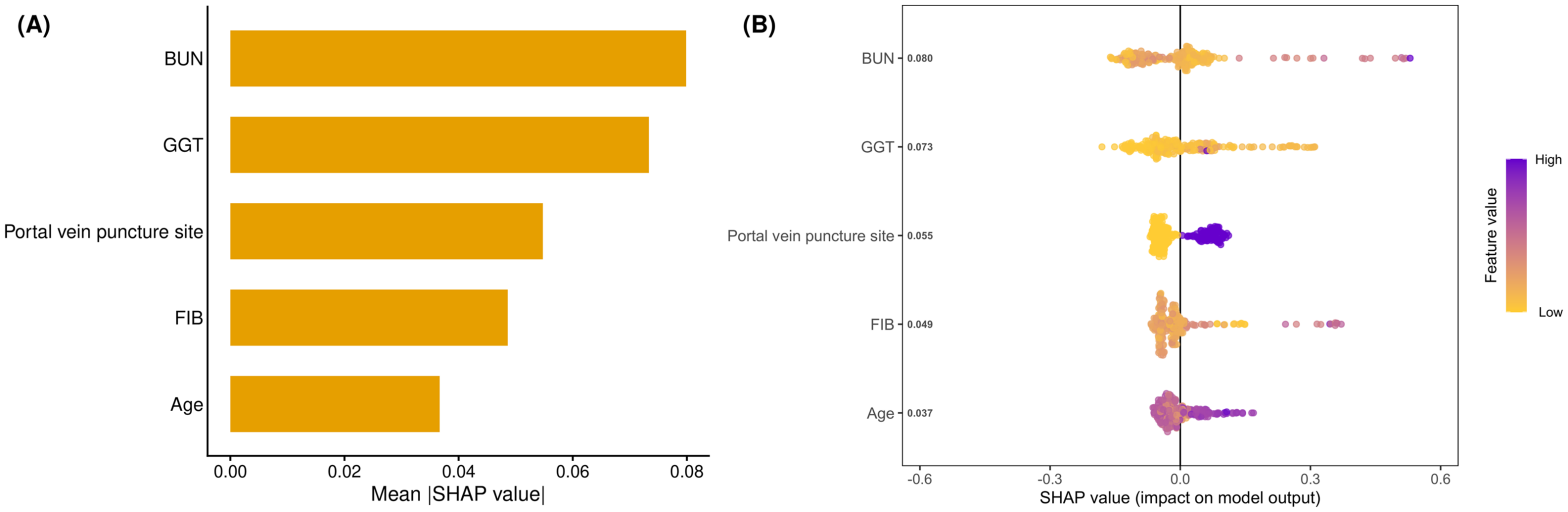
SHAP interpretation of the XGBoost model. **(A)** Feature importance ranking. **(B)** Beeswarm plot displaying the distribution and direction of global feature effects.

To interpret the XGBoost model, SHAP-based feature ranking and beeswarm plots were generated. As shown in Figure 5A, in descending order of mean absolute SHAP value, the five features were BUN, GGT, the puncture site of the portal vein, FIB, and age. In the beeswarm plot, yellow and purple represent lower and higher feature values, respectively. As illustrated in Figure 5B, higher BUN levels were mainly associated with positive SHAP contributions, indicating an increased predicted risk. GGT showed a similar pattern, with higher values corresponding to higher risk estimates. Among surgery-related factors, the puncture site of the portal vein exerted a notable influence on model predictions, with puncture of the right branch presenting more SHAP values in the positive range than the left branch. Elevated FIB values also contributed positively to the prediction. In addition, age demonstrated a positive association with predicted risk, as reflected by progressively increasing SHAP values with advancing age.

## 4 Discussion

This retrospective study included 297 patients with cirrhosis who underwent TIPS at the First Hospital of Shanxi Medical University, of whom 89 developed post-TIPS OHE. Using demographic characteristics, preoperative clinical and laboratory data, and procedure-related variables, five machine learning models were developed and evaluated for risk prediction. The models demonstrated comparable discriminative performance, with only modest differences observed. In the test cohorts, the XGBoost model achieved a slightly higher AUC than the other models and showed a relatively balanced performance in terms of sensitivity and specificity. The DeLong’s test indicated that most pairwise comparisons did not reach statistical significance. Nevertheless, XGBoost exhibited stable performance across multiple evaluation metrics, suggesting its applicability under the data conditions of this study. To further evaluate the robustness of the model, sensitivity analyses using alternative feature sets were conducted, and the results showed similar discrimination and model ordering across models. The calibration curve of the XGBoost model was closer to the ideal line in terms of overall trend, yet some systematic bias remained. DCA indicated that XGBoost provided comparatively greater net benefit within low to intermediate threshold ranges, suggesting potential clinical utility in applicable clinical contexts.

In recent years, machine learning methods have been increasingly used in clinical risk prediction, and they have shown clear advantages in capturing multivariable and nonlinear associations (9). As a gradient-boosting ensemble algorithm, XGBoost has demonstrated consistent discrimination and stable performance in a range of clinical prediction studies (10). Previous evidence has shown that XGBoost models developed using routine clinical and laboratory parameters can effectively predict the risk of hepatocellular carcinoma in patients with metabolic-associated fatty liver disease (11). On the basis of this study’s findings, the XGBoost model is able to generate risk estimates for post-TIPS OHE using routinely collected perioperative parameters. As the model relies solely on standard clinical measurements, individualized risk quantification can be performed preoperatively or during the perioperative period without introducing additional testing requirements. Such estimates may support patient stratification according to predicted risk. For patients identified as having an increased likelihood of OHE, consideration may be given to enhanced postoperative surveillance or to the application of targeted management measures within the scope of current clinical practice.

Based on the SHAP analysis of the XGBoost model, we further examined the key predictors included in the model. The results identified BUN as an important factor associated with the risk of post-TIPS OHE. A multicentre study has similarly reported that BUN is a critical predictor of CHE in patients with cirrhosis (12). As the metabolic end product of urea synthesis, BUN reflects nitrogen metabolism and, to some extent, the efficiency of ammonia clearance. It also serves as a key indicator of renal function and protein catabolism; elevated levels often suggest increased catabolic activity or reduced effective circulating volume, both of which may contribute to HE by increasing ammonia load or impairing the body’s clearance capacity (13). In addition, higher BUN levels have been identified as an independent risk factor related to both the severity and prognosis of OHE (14). Moreover, this study found that elevated GGT levels were positively associated with the risk of post-TIPS HE. GGT is commonly used as a biochemical indicator of hepatocellular injury, cholestasis, and oxidative stress, and increased levels typically reflect heightened hepatic inflammation and oxidative metabolic stress. Previous studies have demonstrated that GGT participates in glutathione metabolism and promotes the generation of reactive oxygen species, thereby playing an important role in oxidative stress and inflammatory processes in the liver and contributing to the development of various liver diseases (15). In addition, elevated GGT has been reported as an independent risk factor for one-year mortality in patients with hepatitis B-related HE (16).

Regarding procedure-related factors, this study found that puncture of the right portal vein branch was associated with the occurrence of post-TIPS OHE. This association may relate to the anatomical characteristics of portal venous inflow. Previous studies have shown that the right portal vein branch receives most of its inflow from the superior mesenteric vein, whereas the left branch predominantly carries splenic venous blood. Because exogenous ammonia is absorbed mainly through the superior mesenteric venous system, creating a shunt via the left portal branch may reduce the amount of ammonia entering the systemic circulation compared with shunting via the right branch (17). However, other work has suggested that the relative contributions of the splenic and superior mesenteric veins are not fixed but may vary with the angulation among the portal, splenic, and mesenteric veins and with their respective flow proportions (18). A randomized controlled trial published in 2025 reported that, in the absence of clear anatomical indications, routine puncture of the left portal vein branch is not recommended, and the choice of puncture site should instead be guided primarily by the patient’s hepatic anatomy and operator experience (19). Consequently, the association observed in this study regarding puncture location should be interpreted as observational, and its underlying mechanisms warrant further validation through hemodynamic and imaging-based investigations.

The SHAP analysis further indicated that higher FIB levels contributed positively to the risk of post-TIPS OHE. As a classic acute-phase reactant, FIB reflects the degree of systemic inflammation. Prior research has shown that inflammatory activation plays a synergistic role in the development of HE by potentiating the neurotoxic effects of hyperammonemia and promoting neuroinflammation, thereby exacerbating cerebral dysfunction (20). This study also identified a significant association between age and the occurrence of post-TIPS HE, which is consistent with previous findings (21). As individuals age, organ function progressively declines, alongside impaired intestinal barrier integrity and reduced ability to clear or tolerate harmful metabolites. These age-related changes collectively increase the risk of developing OHE after TIPS (22).

In this study, although the Child-Pugh and MELD-Na scores are widely used clinical assessments of hepatic function, they did not demonstrate clear predictive value during model training. This may be attributable to the relatively homogeneous distribution of hepatic function within the study population, which limited the discriminative ability of these scores. Furthermore, the principal components of both scores had already been included in the model as individual variables. During feature selection, the model preferentially retained these underlying predictors, and the composite scores did not provide additional incremental value.

Compared with previous studies evaluating the risk of post-TIPS HE, the present work expands upon existing approaches by incorporating more comprehensive model assessment and interpretability analyses. In addition to comparing the discriminative performance of different models, we further examined the calibration of predicted probabilities and evaluated the potential clinical net benefit across varying threshold probabilities using DCA. Moreover, the predictive model was developed using routinely available laboratory parameters and procedure-related factors, and its outputs were interpreted with SHAP analysis to delineate the relative contributions and directional effects of individual predictors within the model.

This study has several limitations. First, this study was a single-center retrospective analysis. The limited completeness of clinical records restricted the inclusion of some potentially relevant variables, such as stent characteristics and nutritional status. In addition, the test cohort was relatively small, and the low number of outcome events may have affected the stability of the model performance estimates and led to a degree of optimism. The study was limited to OHE as the sole endpoint. CHE was not included, and no further assessment of the severity, recurrence, or timing of OHE was performed. Finally, external validation was not performed, and the generalizability of the model remains uncertain. Future studies based on multicenter prospective cohorts, incorporating independent validation and time-to-event analyses, are needed to provide a more comprehensive assessment of HE risk following TIPS.

## 5 Conclusion

This study developed and compared several machine learning models using a single-center cohort of patients with cirrhosis to predict the risk of overt hepatic encephalopathy following TIPS. The findings indicate that models based on routine clinical data demonstrated acceptable discriminatory ability, with XGBoost showing relatively stable performance across multiple evaluation metrics. SHAP analysis further identified BUN, GGT, age, FIB, and the portal vein puncture site as important contributors to the prediction of post-TIPS OHE, suggesting their potential utility in perioperative risk assessment and clinical decision-making.

## Data Availability

The raw data supporting the conclusions of this article will be made available by the authors, without undue reservation.
