# Supplementary material for: Machine learning-based risk prediction of overt hepatic encephalopathy after transjugular intrahepatic portosystemic shunt in patients with cirrhosis: a cohort study
Source: Front Med (Lausanne). 2026 May 29;13:1809634. doi: 10.3389/fmed.2026.1809634 (PMC13259985; doi:10.3389/fmed.2026.1809634)
Supplement: Supplementary file 1 [file Data_Sheet_1.DOCX]

Supplementary Table S1. Sensitivity analysis using Feature Set A (GGT replaced by diabetes)

Performance of the LR, SVM, RF, XGBoost, and ANN models using a predictor set in which diabetes replaced GGT. Reported metrics include AUC (95% CI), balanced accuracy, sensitivity, specificity, and F1 score for both training and test cohorts.

| Models | AUC (95% CI) | Balanced accuracy | Sensitivity | Specificity | F1 score |
| --- | --- | --- | --- | --- | --- |
| Training cohort | |  |  |  |  |
| LR | 0.689 (0.616-0.763) | 0.600 | 0.370 | 0.830 | 0.422 |
| SVM | 0.709 (0.634-0.783) | 0.596 | 0.247 | 0.945 | 0.360 |
| RF | 0.833 (0.779-0.888) | 0.752 | 0.644 | 0.861 | 0.657 |
| XGBoost | 0.731 (0.664-0.799) | 0.644 | 0.397 | 0.891 | 0.483 |
| ANN | 0.807 (0.743-0.871) | 0.756 | 0.658 | 0.855 | 0.662 |
| Test cohort | |  |  |  |  |
| LR | 0.635 (0.474-0.796) | 0.563 | 0.313 | 0.814 | 0.345 |
| SVM | 0.644 (0.496-0.791) | 0.496 | 0.063 | 0.930 | 0.100 |
| RF | 0.676 (0.515-0.837) | 0.594 | 0.375 | 0.814 | 0.400 |
| XGBoost | 0.755 (0.671-0.914) | 0.598 | 0.313 | 0.884 | 0.385 |
| ANN | 0.701 (0.546-0.855) | 0.610 | 0.500 | 0.721 | 0.444 |

Supplementary Table S2. Sensitivity analysis using Feature Set B (GGT and diabetes both included)

Performance of all five models when both GGT and diabetes were included in the predictor set. Metrics reported include AUC (95% CI), balanced accuracy, sensitivity, specificity, and F1 score for both cohorts.

| Models | AUC (95% CI) | Balanced accuracy | Sensitivity | Specificity | F1 score |
| --- | --- | --- | --- | --- | --- |
| Training cohort | |  |  |  |  |
| LR | 0.691 (0.617-0.764) | 0.630 | 0.411 | 0.848 | 0.469 |
| SVM | 0.724 (0.647-0.801) | 0.623 | 0.301 | 0.945 | 0.423 |
| RF | 0.858 (0.807-0.909) | 0.786 | 0.712 | 0.861 | 0.703 |
| XGBoost | 0.776 (0.715-0.837) | 0.695 | 0.603 | 0.788 | 0.579 |
| ANN | 0.847 (0.794-0.900) | 0.744 | 0.603 | 0.885 | 0.647 |
| Test cohort | |  |  |  |  |
| LR | 0.637 (0.476-0.798) | 0.563 | 0.313 | 0.814 | 0.345 |
| SVM | 0.645 (0.476-0.815) | 0.539 | 0.125 | 0.953 | 0.200 |
| RF | 0.701 (0.553-0.848) | 0.614 | 0.438 | 0.791 | 0.438 |
| XGBoost | 0.749 (0.610-0.888) | 0.657 | 0.500 | 0.814 | 0.500 |
| ANN | 0.674 (0.518-0.831) | 0.583 | 0.375 | 0.791 | 0.387 |

Supplementary Table S3. Mean AUC and Standard Deviation of Each Model Across Repeated Cross-Validation

Performance of the LR, SVM, RF, XGBoost, and ANN models using 5-fold repeated cross-validation (10 repetitions) on the full dataset. Reported metrics include the mean AUC and standard deviation (SD) across all resampling iterations.

| Model | Mean AUC | SD |
| --- | --- | --- |
| LR | 0.633 | 0.059 |
| SVM | 0.601 | 0.077 |
| RF | 0.638 | 0.078 |
| ANN | 0.610 | 0.080 |
| XGBoost | 0.659 | 0.103 |
